# Socioeconomic status and self-reported, screen-detected and total diabetes prevalence in Chinese men and women in 2011-2012: a nationwide cross-sectional study

**DOI:** 10.7189/jogh.08.020501

**Published:** 2018-12

**Authors:** Hongjiang Wu, Caroline A Jackson, Sarah H Wild, Weiyan Jian, Jianqun Dong, Danijela Gasevic

**Affiliations:** 1 Usher Institute of Population Health Sciences and Informatics, University of Edinburgh, UK; 2 School of Public Health, Peking University, Beijing, China; 3 National Centre for Chronic and Noncommunicable Disease Control and Prevention, Chinese Centre for Disease Control and Prevention, Beijing, China

## Abstract

**Background:**

A rapid epidemiological transition is taking place in China and the association between socioeconomic status (SES) and diabetes prevalence is not clear and may vary by population characteristics and geography within the country. We describe the associations between educational level, annual household living expenditure (AHLE) and diabetes prevalence in a large middle-aged and elderly Chinese population using data from a nationwide cross-sectional study.

**Methods:**

We used data from the China Health and Retirement Longitudinal Study, which collected information from interviews and blood tests from a nationwide sample of people over 44 years of age in 2011-2012. We used multivariable logistic regression to describe the association between highest levels of education (high school or above compared to illiterate) or AHLE (top vs bottom quartile) and self-reported, screen-detected or total diabetes prevalence. We stratified by sex and adjusted for age, education or AHLE (as appropriate), urban, rural or migrant residence status and geographical area.

**Results:**

Complete data were available for 10 100 participants of whom 10.5% and 28.9% had the highest and the lowest levels of education respectively. Overall prevalence of self-reported diabetes was 6.0% and of screen-detected diabetes was 9.8%. Higher education level was associated with both self-reported diabetes (odds ratio (OR) = 2.41, 95% confidence interval CI = 1.36-4.46) and total diabetes (OR = 1.53 95%, CI = 1.10-2.15) only in men. AHLE was associated with self-reported diabetes in men (OR = 1.87, 95% CI = 1.26-2.84) and women (OR = 2.31, 95% CI = 1.62-3.34). There was no association between SES and screen-detected diabetes for men or women.

**Conclusions:**

SES inequalities exist in prevalence of diabetes in China and can be used to inform approaches to prevention. Identification and appropriate intervention for people with undiagnosed diabetes is required for all SES groups.

As a result of rapid economic development, urbanization and aging, China is facing a high and increasing burden of diabetes [1]. The overall prevalence of diabetes (both diagnosed and undiagnosed) in China is estimated to have increased from 2.5% to 10.9% between 1994 and 2013 [2,3]. People with diabetes are at a higher risk of long-term damage, dysfunction and failure of various organs (eg, heart, eye, kidney and nerves) than those without diabetes [4]. As the early stages of type 2 diabetes are asymptomatic, a large proportion of diabetes is undiagnosed in many countries.

Globally, the prevalence of diabetes is strongly related to socioeconomic status (SES), with different strengths and directions of the association observed in different populations. Studies from developed countries report an inverse association between SES and prevalence of diabetes [5, 6], while the opposite has been found in some developing countries [7,8]. Previous studies on diabetes prevalence in China generally considered SES as a descriptive variable of the study sample or a potential confounder of the relationship between other variables and health outcomes [9]. The few studies in China that investigated the association between SES and diabetes prevalence as their primary aim were based on small geographical areas and have given inconsistent results [10-14]. In addition, none of the studies above has explored in detail the association between SES and prevalence of all self-reported, screen-detected, and total (combined self-reported and screen-detected) diabetes. The strengths and directions of SES inequalities in self-reported and screen-detected diabetes might differ as a consequence of differences in awareness of diabetes or in access to health care associated with SES [12]. Among Chinese adults who have diabetes diagnosed by fasting plasma glucose, 2-hour plasma glucose, haemoglobin A_1c_ (HbA_1c_) or a self-reported history of diabetes according to American Diabetes Association (ADA) 2010 criteria in the China national diabetes survey in 2013, only about 36% reported a previous diagnosis of diabetes [3]. However, the evidence for the association between SES and awareness of diabetes in China is still sparse.

Therefore, the main aim of this study is to describe the association between two measures of SES and self-reported, screen-detected and total diabetes prevalence in a nationwide sample of the middle-aged and older population of China. In addition, we describe the association between SES and awareness of diabetes.

## METHODS

### Study population and data collection

We used data from the baseline survey of the population-based China Health and Retirement Longitudinal Study (CHARLS), a nationally representative longitudinal survey of the mainland Chinese population of adults aged 45 years or older, to conduct a cross-sectional study. Details of the CHARLS have been described in detail elsewhere [15]. Briefly, a total of 17 708 respondents from 10 257 households were recruited in the baseline survey from 150 counties within 28 provinces in mainland China between June 2011 and March 2012. Samples were selected using a multistage probably-proportional-to-size sampling technique, stratified by regions and then by urban districts or rural counties, and by per capita gross domestic product. The response rate for the survey was 80.5%.

Face-to-face interviews were used to collect information on socio-demographic characteristics (including age at the baseline survey, sex, residence, geographical area of China), and self-reported diabetes status. Venous blood samples were collected by medically-trained staff from the China Centre for Diseases Control and Prevention on the subset of participants who were willing to donate venous blood samples. Participants were asked to fast overnight before blood collection. However, blood was also taken if they had not fasted and their fasting status was recorded. Blood samples were assayed at the Youanmen Centre for Clinical Laboratory of the Capital Medical University in Beijing. Glucose was measured using an enzymatic colorimetric test, and HbA_1c_ was analysed using boronate affinity chromatography. The study protocol was approved by ethical review board of Peking University and written informed consent was obtained from all study participants.

A blood sample was collected for 11 847 (67%) participants. We excluded participants without complete data and those aged less than 45 years. After these exclusions, 10 100 participants remained for the final analyses.

### Assessment of exposure, outcome and main variables

In the present study, the exposure of interest is participants’ SES, as measured by the highest educational level attained and annual household living expenditure (AHLE). Due to the small number of participants in some levels of original education categorization in CHARLS, we categorized education into five groups: illiterate (no formal education), literate (did not finish primary school but capable of reading or writing, or finish Sishu), elementary school, middle school, and high school and above (high school, vocational school, associate degree, bachelor degree, master degree and PhD). Sishu is a historical Chinese educational tradition for children that provided private tutorials for basic education. Sishu was formally abolished in 1905 in China, but a very small proportion of participants in CHARLS reported that their highest educational level was Sishu. These participants were included in the ‘literate’ group. We categorized the continuous AHLE variable into quartiles with quartile 1 (Q1) including the lowest 25% and quartile 4 (Q4) the highest 25%. We used AHLE as a proxy for household income in this study for two reasons: 1) the number of missing values for household income was substantial, and 2) household living expenditure patterns are generally more stable than income over time, suggesting that this may be a more reliable measure of SES that better reflects the economic well-being of households than household income [16].

The main outcomes were self-reported diabetes, screen-detected diabetes, and total diabetes. Participants were asked: “Have you ever been diagnosed with diabetes or high blood sugar by a doctor?”, with those reporting “yes” defined as having self-reported diabetes. Those who responded “no” but had a fasting plasma glucose ≥7.0 mmol/L (126 mg/dL), random plasma glucose ≥11.1 mmol/L (200 mg/dL) or HbA_1c_ value ≥6.5% were defined as having screen-detected diabetes. Total diabetes prevalence was defined as the presence of either self-reported or screen-detected diabetes. The secondary outcome was awareness of diabetes defined as the proportion of people with either screen-detected or self-reported diabetes that had self-reported diabetes.

We defined type of residence as urban, rural and migrant. Migrants were living in urban areas but who had an agricultural hukou. Hukou is China’s permanent residence administrative registration system, which determines where citizens are allowed to live. Migrants to urban areas are commonly born and raised in rural areas and are severely disadvantaged compared to permanent urban residents in terms of access to health care, education, housing benefits and basic infrastructure [17]. Geographical areas of China were categorized into four groups: West China, East China, Central China and Northeast China. However, due to the small number of participants in Northeast China, we combined Central China and Northeast China as one group as they are at a similar level of economic development [18].

### Statistical analysis

We calculated age-standardised prevalence of self-reported, screen-detected, and total diabetes in men and women using the direct method of standardisation with the total population of CHARLS data by 10-year age groups as the standard population. We compared baseline characteristics of participants across categories of education and AHLE using χ^2^ tests for categorical data. We used univariable and multivariable logistic regression models to obtain odds ratios and 95% CIs for the association between SES and prevalence of self-reported, screen-detected and total diabetes, and for the association between SES and awareness of diabetes, stratifying by sex. We adjusted for age, education or AHLE (as appropriate), residence and geographical area in the multivariable models. We used likelihood ratio tests to assess the multiplicative interaction between SES and all other included variables, by comparing models with and without interaction terms. We carried out a sensitivity analysis for the association between SES and self-reported diabetes prevalence in the population who had complete data regardless of availability of blood sample. We compared the age-standardised prevalence of self-reported diabetes in participants used for primary and sensitivity analyses, and compared the characteristics of participants included in and excluded from our primary analyses. We performed analyses using R software version 3.3.2 (R Foundation for Statistical Computing, Vienna, Austria).

## RESULTS

Of the 10 100 participants included in the primary analysis, 47.4% (n = 4791) were men. The mean age was 60 (SD = 9.4) years for men and 59 (SD = 9.7) years for women. Both men and women in the highest education category were younger, more likely to live in urban areas and less likely to live in West China than participants with lower educational levels (**Table 1**). The patterns of co–variates by AHLE were generally similar to those for education. However, AHLE was not significantly associated with the geographical area of China.

**Table 1:** Characteristics of included CHARLS participants by sex and categories of education and annual household living expenditure (n = 10 100)*

Characteristics	Education						Annual household living expenditure				
	**Illiterate**	**Literate**	**Elementary school**	**Middle school**	**≥High schoole**	***P*-value**	**Q1**	**Q2**	**Q3**	**Q4**	***P*-value**
**Men** (n = 4791)	658 (13.7)	926 (19.3)	1291 (26.9)	1243 (25.9)	673 (14.0)		1179 (24.6)	1201 (25.1)	1204 (25.1)	1207 (25.2)	
**Age** (years)	67 (59-74)	61 (56-69)	61 (55-67)	55 (49-61)	54 (49-61)		62 (56-70)	60 (54-67)	59 (53-65)	56 (49-63)	
**Residence:**						<0.0001					<0.0001
Rural	532 (80.9)	657 (71.0)	902 (69.9)	749 (60.3)	294 (43.7)		880 (74.6)	840 (69.9)	762 (63.3)	652 (54.0)	
Migrants	98 (14.9)	200 (21.6)	252 (19.5)	251 (20.2)	99 (14.7)		177 (15.0)	208 (17.3)	237 (19.7)	278 (23.0)	
Urban	28 (4.3)	69 (7.5)	137 (10.6)	243 (19.5)	280 (41.6)		122 (10.3)	153 (12.7)	205 (17.0)	277 (22.9)	
**Geographical area:**						<0.0001					0.31
West	257 (39.1)	355 (38.3)	463 (35.9)	413 (33.2)	191 (28.4)		400 (33.9)	417 (34.7)	438 (36.4)	424 (35.1)	
Central and northeast	246 (37.4)	300 (32.4)	443 (34.3)	480 (38.6)	280 (41.6)		415 (35.2)	462 (38.5)	437 (36.3)	435 (36.0)	
East	155 (23.6)	271 (29.3)	385 (29.8)	350 (28.2)	202 (30.0)		364 (30.9)	322 (26.8)	329 (27.3)	348 (28.8)	
**Women** (n = 5309)	2256 (42.5)	942 (17.7)	932 (17.6)	791 (14.9)	388 (7.3)		1300 (24.5)	1352 (25.5)	1324 (24.9)	1333 (25.1)	
**Age** (years)	61 (56-70)	58 (53-63)	57 (49-64)	51 (48-58)	51 (48-56)		62 (55-70)	58 (52-65)	57 (50-63)	55 (48-61)	
**Residence:**						<0.0001					<0.0001
Rural	1717 (76.1)	632 (67.1)	567 (60.8)	371 (46.9)	92 (23.7)		936 (72.0)	904 (66.9)	827 (62.5)	712 (53.4)	
Migrants	427 (18.9)	227 (24.1)	218 (23.4)	177 (22.4)	59 (15.2)		233 (17.9)	260 (19.2)	295 (22.3)	320 (24.0)	
Urban	112 (5.0)	83 (8.8)	147 (15.8)	243 (30.7)	237 (61.1)		131 (10.1)	188 (13.9)	202 (15.3)	301 (22.6)	
**Geographical area:**						<0.0001					0.27
West	806 (35.7)	295 (31.3)	326 (35.0)	230 (29.1)	117 (30.2)		414 (31.8)	445 (32.9)	464 (35.0)	451 (33.8)	
Central and northeast	775 (34.4)	373 (39.6)	375 (40.2)	331 (41.8)	163 (42.0)		483 (37.2)	536 (39.6)	499 (37.7)	499 (37.4)	
East	675 (29.9)	274 (29.1)	231 (24.8)	230 (29.1)	108 (27.8)		403 (31.0)	371 (27.4)	361 (27.3)	383 (28.7)	

Q – quarter

*Values are N (%): % are row% for sex and column% for other variables; age is shown as median with interquartile range.

**Figure 1:**
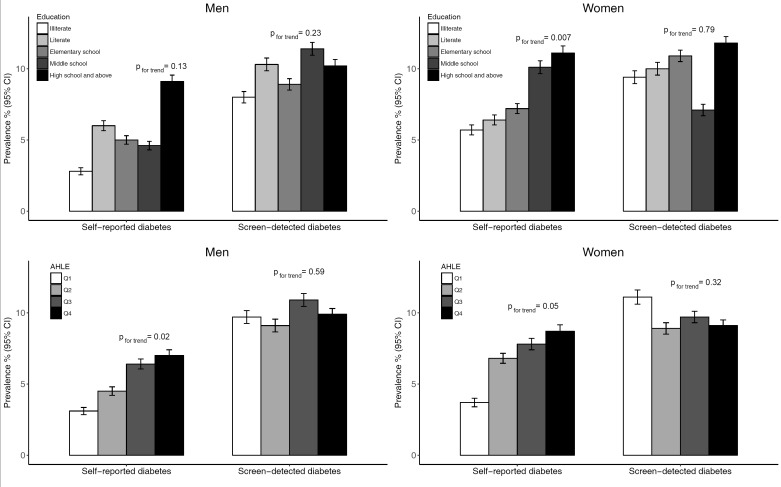
Age-standardised prevalence and 95% confidence intervals (CI) of self-reported and screen-detected diabetes by education and AHLE for men and women.

Overall, 1592 participants were found to have diabetes, of whom 62.0% (987) had screen-detected diabetes. After age-standardisation, the prevalence of self-reported diabetes was 5.2% (95% CI = 4.9%-5.5%) for men and 6.6% (95% CI = 6.3%-7.0%) for women, and screen-detected diabetes was identified in a further 10.1% (95% CI = 9.7%-10.6%) of men and 9.5% (95% CI = 9.1%-10.0%) of women (Table S1 in **Online Supplementary Document(Online Supplementary Document)**). Generally, the age-standardised prevalence of self-reported diabetes and total diabetes were higher in men and women with higher educational level and AHLE level (**Figure 1** and Table S1 in **Online Supplementary Document(Online Supplementary Document)**). However, there was no clear association between education or AHLE and prevalence of screen-detected diabetes in either sex. Men and women living in urban areas had the highest prevalence of self-reported diabetes and total diabetes compared to rural and migrant populations, and those living in East China and West China had the highest prevalence of self-reported diabetes and screen-detected diabetes, respectively. The age-standardised awareness rate of diabetes was highest in men with the highest educational level, in both men and women with the highest AHLE level and those living in urban areas and East China.

**Figure 2:**
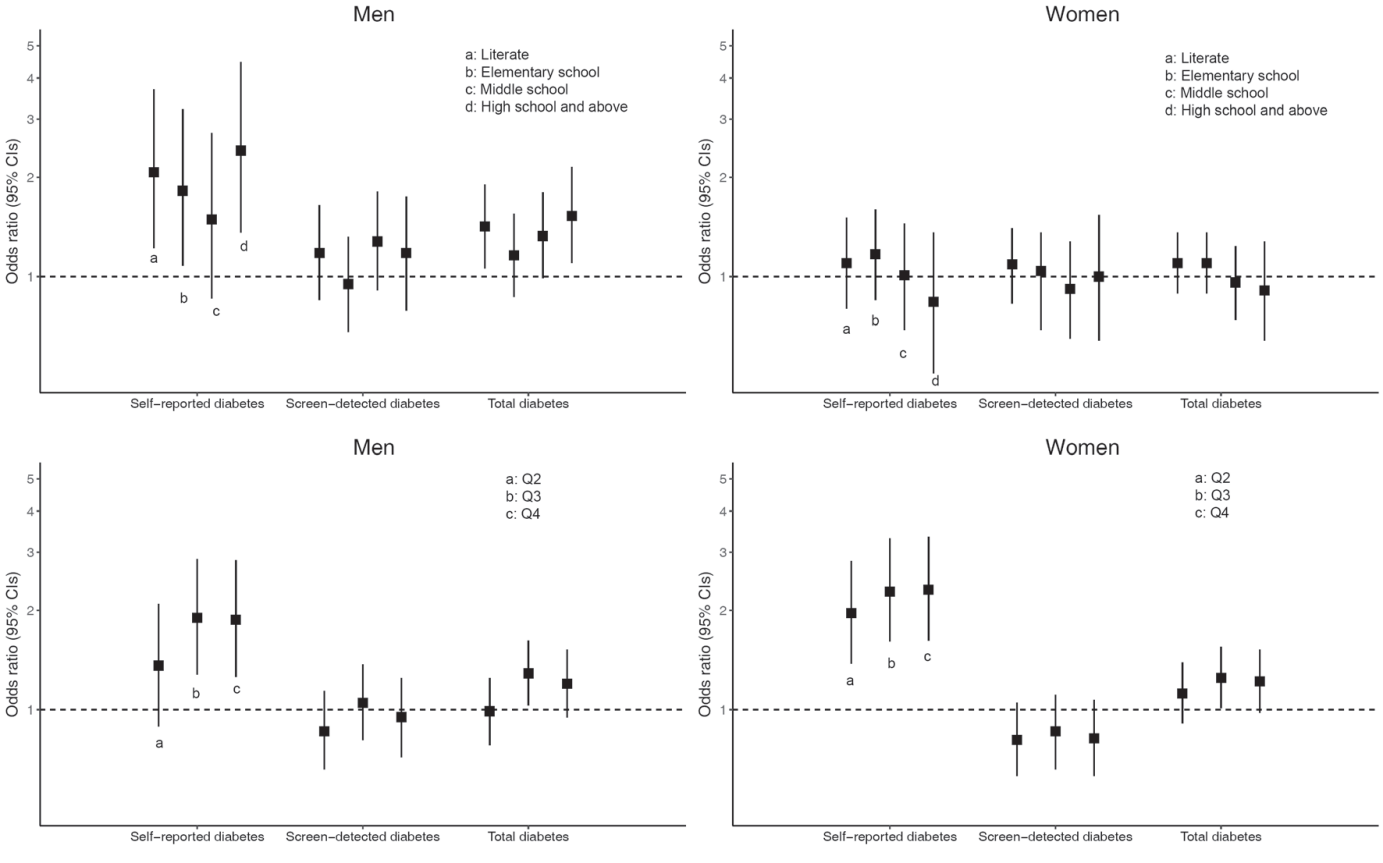
Sex-specific adjusted odds ratios and 95% confidence intervals (CI) for prevalence of different components of diabetes according to education with comparison to the illiterate group, and annual household living expenditure with comparison to the lowest quartile.

After adjustment for age, AHLE, residence and geographical area, higher educational level was statistically significantly associated with increased odds of both self-reported diabetes and total diabetes compared to the illiterate group in men, but not in women (**Figure 2** and Table S2 in **Online Supplementary Document(Online Supplementary Document)**). The odds ratio of having self-reported diabetes and total diabetes was 2.41 (95% CI = 1.36-4.46) and 1.53 (95% CI = 1.10-2.15) for men with at least high school education compared to those who were illiterate. Higher AHLE was also significantly associated with increased odds of self-reported diabetes compared to the lowest quartile of AHLE in both men and women (**Figure 2** and Table S3 in **Online Supplementary Document(Online Supplementary Document)**). The odds ratio of having self-reported diabetes was 1.87 (95% CI = 1.26-2.84) for men and 2.31 (95% CI = 1.62-3.34) for women in the highest AHLE quartile compared to those in the lowest AHLE quartile. However, a statistically significant positive association between AHLE and total diabetes was only observed in the third quartile (Q3) category compared to the lowest AHLE in both men (OR = 1.29 95% CI = 1.03–1.62) and women (OR = 1.25 95% CI = 1.01–1.55). Neither educational level nor AHLE was associated with screen-detected diabetes.

**Figure 3:**
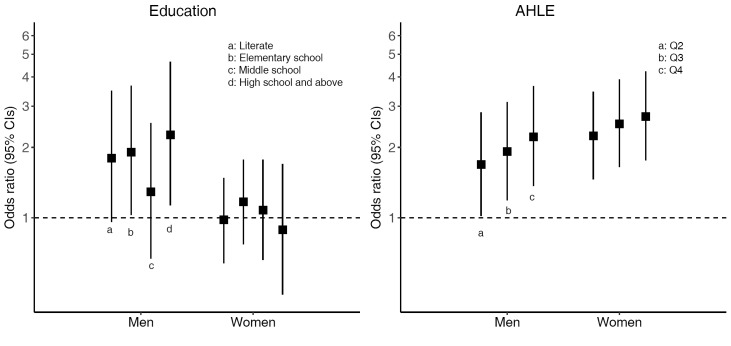
Sex-specific adjusted odds ratios and 95% confidence intervals (CI) for awareness of diabetes according to education with comparison to the illiterate group, and annual household living expenditure with comparison to the lowest quartile.

Among people with diabetes, both educational level (only in men) and AHLE were positively associated with awareness of diabetes (**Figure 3**). Compared to the lowest educational level, the odds ratios of awareness of diabetes with at least high school education were 2.26 (95% CI = 1.13-4.64) and 0.89 (95% CI = 0.47-1.70) for men and women, respectively. Compared to the lowest AHLE quartile, the odds ratios of awareness of diabetes in the highest AHLE quartile were 2.22 (95% CI = 1.37-3.65) and 2.71 (95% CI = 1.76-4.22) for men and women, respectively. There was no evidence for interaction between education or AHLE and age, AHLE or education (as appropriate), residence and geographical area on diabetes prevalence or awareness of diabetes.

In sensitivity analyses, we determined the association between SES and self-reported diabetes using the population regardless of whether blood samples were available for participants to explore potential for selection bias. The results were similar to that from the population included in our primary analyses (Table S4 and Table S5 in **Online Supplementary Document(Online Supplementary Document)**). The age-standardised prevalence of self-reported diabetes in participants for sensitivity analyses was 5.2% (95% CI = 4.7%-5.7%) for men and 6.3% (95% CI = 5.8%-6.9%) for women, similar to the estimates for the participants for our primary analyses among the sub-group of people for whom blood test results and other complete data were available. However, there were significant differences in characteristics between the participants included and excluded in our primary analyses. Participants excluded from the analyses because they did not provide a blood sample or had missing data, were younger, more educated, and were more likely to live in urban and East China compared to those included, but there were no differences in sex, AHLE and proportion of self-reported diabetes (Table S6 in **Online Supplementary Document(Online Supplementary Document)**).

## DISCUSSION

Using population-based cross-sectional data from the CHARLS for 2011-2012, we found that men with a higher level of education, and both men and women with a higher level of AHLE had higher odds of having both self-reported diabetes and total diabetes prevalence, and of being aware of having diabetes after controlling for confounding variables. However, there was no evidence of a significant association between education or AHLE and screen-detected diabetes. These associations were robust across different residence categories and geographical areas of China.

The findings of this study are consistent with previous studies in China reporting a positive association between educational level [13], household income [13], individual income [14] and self-reported diabetes. However, they are inconsistent with other studies reporting a non-significant or inverse association between educational level or income and self-reported, screen-detected or total diabetes [10-12,14]. Heterogeneity of study populations (eg, age distribution), study location, classification and definition of education and income, and adjustments for variables may have led to the discrepant results between studies.

We found that people with higher SES in China were more likely to have self-reported diabetes, which was consistent with findings from other developing countries [7,8], but inconsistent with those reported from developed countries [5,6]. One possible explanation for the increased prevalence of self-reported diabetes in high SES groups may be due to the higher awareness of diabetes in high SES groups. Findings in this study as well as previous studies in China showed people with diabetes of higher SES were more likely to be aware of their diabetes status compared to people of lower SES [12,19]. This might be because people with higher SES have greater access to health care, such as routine health checks, than people with lower SES. The early stages of diabetes are usually asymptomatic and the disease may be sub-clinical for many years. Thus diabetes may remain undiagnosed for a long period of time, until blood glucose tests are performed or complications occur [4].

The association between education and self-reported and total diabetes was only significant in men but not women. The reason for this sex difference is unclear but may be related to the smaller proportions of women who completed higher levels of education. Education may have a different value and implications for men and women. Middle-aged and older Chinese women grew up prior to the recent economic development and a high education may not be necessary for the work and life. Therefore, education may not provide a useful measure of SES of middle-aged and older Chinese women. Though education practices have varied over time in China, we found no evidence of modification of the association between education and diabetes prevalence by birth cohort. In addition, the reason may also be that women are at an earlier stage of epidemiological transition in the association between education and diabetes prevalence shifting from being positive to inverse [20].

A previous study from an urban area of China found that SES was inversely associated with prevalence of screen-detected diabetes [10]. We found similar prevalence of screen-detected diabetes across all SES groups. In our study, the prevalence of screen-detected diabetes was much higher than self-reported diabetes across all SES groups and all age categories, which was consistent with the pattern observed in the previous China national diabetes survey [3]. The proportion of total diabetes that was self-reported in our study was about 38%, similar to that in the national diabetes survey in 2013 (36%). This suggests that a very high proportion of Chinese population would have screen-detected diabetes suggesting that population-based programmes to increase the awareness of risk factors, consideration of the potential costs and benefits of early detection and diagnosis of diabetes are needed across all levels of SES.

The likelihood of developing diabetes depends upon risk factor patterns. Diabetes is a chronic disease highly related to lifestyle behaviours [21]. Rapid income growth in China is adversely affecting the Chinese diet, with dietary patterns shifting from a traditional Chinese healthy diet toward an increased consumption of high energy foods [22,23]. Furthermore, this transition is occurring faster among poor people than among affluent groups [22]. In addition, according to the China Health and Nutrition Survey from 1991 to 2011, there was a significant decline in physical activity in the Chinese population, especially for occupational activity [24]. This study also found higher education and income levels were associated with lower levels of physical activity. As these behavioural risk factors for diabetes are strongly patterned by SES in China, these are likely to contribute to differences in diabetes prevalence. In addition to lifestyle behaviours, the effect of SES on diabetes, particularly the proportion that is screen-detected may also be influenced by access to health care [25].

Some limitations of our study should be recognized. First, although CHARLS is a nationally representative study, we excluded a large number of participants who did not provide a blood sample or had missing data. The participants excluded had different characteristics from those included in our primary analyses, indicating that voluntary response bias may be introduced in this process and the participants included in the primary analyses may not be representative of the entire Chinese population aged 45 years or older in China. Second, as most of the data in this study were self-reported, information bias is possible. For example, AHLE is considered as sensitive information, thus the data may be inaccurate. Third, screen-detected diabetes is based on a single test that may overestimate the true prevalence of newly diagnosed diabetes. Both the World Health Organization and ADA recommend that clinical diagnosis of diabetes should be made on the basis of two abnormal test results in people without symptoms of hyperglycaemia [4,26]. In addition, information on important potential mediating factors between SES and diabetes was incomplete or not collected, such as BMI and physical activity that are risk factors for diabetes and whose distribution is also likely to differ by SES [24,27-29]. As a result, we sought to determine whether SES is associated with diabetes, irrespective of the mechanism. Furthermore, the data used in this analysis were collected in 2011–2 and may not represent current patterns given the rapid development in China. Besides, since this is a cross-sectional study, we cannot conclude that the observed association between SES and diabetes is causal. Lastly, the scope of our study did not include people aged less than 45 years. Given the growing number of people with young-onset diabetes, future studies are needed to clarify the association between SES and diabetes prevalence in young Chinese people.

Despite the limitations, to our knowledge this is the first study to assess the association between SES and all self-reported diabetes, screen-detected diabetes, and total diabetes in China, with consideration of geographical and urban-rural differences. The previous studies were conducted in a single city [10,13,14] or a small rural area [11,12] in China, which limited the ability to generalise the results to other areas of China. The participants in our study were selected from a nationwide survey of the Chinese population, which provided a more comprehensive picture of SES associated with prevalence of diabetes in China. In addition, it is important to note that previous studies have adjusted for both body mass index (BMI) and physical activity [10,11,13,14] or only overweight/obesity [12] as potential confounding variables. This may contribute to the many non-significant associations between SES and diabetes prevalence reported by previous studies, if the socioeconomic disparities in diabetes prevalence is mediated by BMI or physical activity gradients across categories of SES [24,27-29].

In conclusion, our results indicate that education in men and a proxy measure of income in both sexes is positively associated with self-reported and total diabetes prevalence and awareness of diabetes in a Chinese population aged 45 years or older. Prevention and monitoring strategies for diabetes prevalence should be developed in China and identification and appropriate intervention for people with undiagnosed diabetes is required for all SES groups. Well-designed prospective cohort studies are needed to describe the association between SES and diabetes incidence and prevalence and to identify the role of diet, physical activity and BMI.
