# Supplementary material for: Socioeconomic status and self-reported, screen-detected and total diabetes prevalence in Chinese men and women in 2011-2012: a nationwide cross-sectional study
Source: J Glob Health. 2018 Jul 29;8(2):020501. doi: 10.7189/jogh.08.020501 (PMC6076585; doi:10.7189/jogh.08.020501)
Supplement: Online Supplementary Document [file jogh-08-020501-s001.pdf]

Online Supplementary Document

Wu et al. Socioeconomic status and self-reported, screen-detected and total diabetes prevalence in Chinese men and women in 2011-2012: a nationwide cross-sectional study

J Glob Health 2018;8:020501

Table S1. Age-standardised* prevalence and 95% CIs of diabetes by sex (n=10100)

Characteristics	Men (n=4791)			Women (n=5309)		
		Diabetes (%)			Diabetes (%)	
	n	Self-reported (n=252)	Screen-detected (n=484)	n	Self-reported (n=353)	Screen-detected (n=503)
Age† (years)						
45-54	1492	4.7 (3.7-5.9)	9.5 (8.1-11.1)	1924	4.4 (3.6-5.4)	7.8 (6.7-9.1)
55-64	1870	5.7 (4.8-6.9)	9.9 (8.7-11.4)	2023	8.2 (7.1-9.5)	9.8 (8.6-11.2)
65-74	1032	5.1 (3.9-6.7)	10.1 (8.4-12.1)	936	8.7 (7.0-10.6)	10.6 (8.8-12.7)
≥75	397	5.5 (3.7-8.2)	13.1 (10.1-16.8)	426	4.9 (3.2-7.4)	12.9 (10.1-16.4)
Education						
Illiterate	658	2.8 (2.6-3.1)	8.0 (7.6-8.4)	2256	5.7 (5.4-6.1)	9.4 (8.9-9.8)
Literate	926	6.0 (5.7-6.4)	10.3 (9.9-10.8)	942	6.4 (6.0-6.7)	10.0 (9.6-10.5)
Elementary school	1291	5.0 (4.7-5.3)	8.9 (8.5-9.3)	932	7.2 (6.9-7.6)	10.9 (10.5-11.3)
Middle school	1243	4.6 (4.3-4.9)	11.4 (11.0-11.9)	791	10.1 (9.7-10.6)	7.1 (6.7-7.5)
High school and above	673	9.1 (8.6-9.5)	10.2 (9.8-10.7)	388	11.1 (10.6-11.6)	11.8 (11.3-12.2)
AHLE						
Q1	1179	3.1 (2.8-3.3)	9.7 (9.3-10.2)	1300	3.7 (3.4-4.0)	11.1 (10.6-11.6)
Q2	1201	4.5 (4.2-4.8)	9.1 (8.7-9.6)	1352	6.8 (6.5-7.2)	8.9 (8.5-9.3)

Q3	1204	6.4 (6.1-6.8)	10.9 (10.4-11.3)	1324	7.8 (7.4-8.2)	9.7 (9.3-10.1)
Q4	1207	7.0 (6.6-7.4)	9.9 (9.5-10.3)	1333	8.7 (8.3-9.2)	9.1 (8.7-9.5)
Residence						
Rural	3134	3.9 (3.6-4.2)	10.0 (9.6-10.5)	3379	5.5 (5.1-5.8)	9.0 (8.6-9.4)
Migrants	900	5.7 (5.3-6.0)	9.4 (9.0-9.8)	1108	6.8 (6.5-7.2)	11.3 (10.9-11.8)
Urban	757	10.1 (9.6-10.5)	11.1 (10.7-11.6)	822	10.9 (10.5-11.4)	9.2 (8.8-9.7)
Geographical area						
West	1679	4.1 (3.8-4.4)	11.4 (10.9-11.9)	1774	4.6 (4.3-4.9)	10.9 (10.5-11.4)
Central and northeast	1749	5.3 (5.0-5.6)	10.1 (9.6-10.5)	2017	7.2 (6.8-7.6)	9.2 (8.7-9.6)
East	1363	6.4 (6.1-6.8)	8.7 (8.3-9.1)	1518	8.3 (7.9-8.7)	8.3 (7.9-8.7)

*age-standardised prevalence according to total population of CHARLS; †crude prevalence

Table S2. Sex-specific unadjusted and adjusted odds ratios (95% CIs) for prevalence of different components of diabetes according to education with comparison to the illiterate group (n=10100)

Diabetes	Education (ref: illiterate)							
	Literate	p value	Elementary school	p value	Middle school	p value	High school and above	p value
Men (n=4791)	926		1291		1243		673	
Self-reported diabetes (n=252)	53		66		56		59	
Model 1	2.16 (1.28-3.82)	0.006	1.92 (1.15-3.35)	0.02	1.68 (1.00-2.96)	0.06	3.42 (2.03-6.02)	<0.0001
Model 2	2.07 (1.22-3.69)	0.01	1.82 (1.08-3.22)	0.03	1.49 (0.86-2.72)	0.17	2.41 (1.36-4.46)	0.004
Screen-detected diabetes (n=484)	101		113		136		69	
Model 1	1.12 (0.81-1.56)	0.51	0.88 (0.64-1.21)	0.42	1.12 (0.82-1.54)	0.47	1.04 (0.73-1.49)	0.82
Model 2	1.18 (0.85-1.65)	0.33	0.95 (0.68-1.32)	0.75	1.28 (0.91-1.81)	0.15	1.18 (0.79-1.75)	0.42
Total diabetes (n=736)	154		179		192		128	
Model 1	1.38 (1.04-1.85)	0.03	1.12 (0.85-1.48)	0.44	1.27 (0.96-1.68)	0.10	1.63 (1.21-2.20)	0.001
Model 2	1.42 (1.06-1.90)	0.02	1.16 (0.87-1.55)	0.32	1.33 (0.99-1.80)	0.06	1.53 (1.10-2.15)	0.01
Women (n=5309)	942		932		791		388	
Self-reported diabetes (n=353)	63		68		57		28	
Model 1	1.11 (0.81-1.50)	0.51	1.22 (0.90-1.64)	0.20	1.20 (0.87-1.65)	0.26	1.20 (0.77-1.81)	0.39

Model 2	1.10 (0.80-1.51)	0.54	1.17 (0.85-1.60)	0.34	1.01 (0.69-1.45)	0.97	0.84 (0.51-1.36)	0.49
Screen-detected diabetes (n=503)	94		89		62		33	
Model 1	1.00 (0.77-1.29)	0.99	0.95 (0.73-1.23)	0.71	0.77 (0.57-1.02)	0.08	0.84 (0.56-1.21)	0.37
Model 2	1.09 (0.83-1.40)	0.54	1.04 (0.79-1.36)	0.77	0.92 (0.66-1.28)	0.64	1.00 (0.64-1.54)	0.98
Total diabetes (n=856)	157		157		119		61	
Model 1	1.05 (0.85-1.28)	0.66	1.06 (0.86-1.30)	0.58	0.93 (0.74-1.16)	0.51	0.98 (0.72-1.30)	0.87
Model 2	1.10 (0.89-1.36)	0.36	1.10 (0.89-1.36)	0.38	0.96 (0.74-1.24)	0.75	0.91 (0.64-1.28)	0.59

Mode l: crude odds ratio; Model 2: adjusted for age, education/AHLE, residence and geographical area

Table S3. Sex-specific unadjusted and adjusted odds ratios (95% CIs) for prevalence of different components of diabetes according to annual household living expenditure with comparison to the lowest quartile (n=10100)

**Diabetes**	**Annual household living expenditure (ref: Q1)**					
	**Q2**	**p value**	**Q3**	**p value**	**Q4**	**p value**
Men (n=4791)	1201		1204		1207	
Self-reported diabetes (n=252)	54		77		82	
Model 1	1.38 (0.91-2.11)	0.14	2.00 (1.36-2.99)	0.0005	2.13 (1.45-3.18)	0.0001
Model 2	1.36 (0.89-2.09)	0.15	1.90 (1.28-2.86)	0.002	1.87 (1.26-2.84)	0.002
Screen-detected diabetes (n=484)	110		132		119	
Model 1	0.87 (0.66-1.13)	0.30	1.06 (0.82-1.37)	0.68	0.94 (0.72-1.23)	0.64
Model 2	0.86 (0.66-1.14)	0.30	1.05 (0.81-1.37)	0.72	0.95 (0.72-1.25)	0.69
Total diabetes (n=736)	164		209		201	
Model 1	0.99 (0.79-1.25)	0.95	1.32 (1.06-1.65)	0.02	1.25 (1.00-1.57)	0.05
Model 2	0.99 (0.78-1.25)	0.93	1.29 (1.03-1.62)	0.03	1.20 (0.95-1.52)	0.13
Women (n=5309)	1352		1324		1333	
Self-reported diabetes (n=353)	94		102		107	

Model 1	1.87 (1.32-2.67)	<0.0001	2.09 (1.48-2.97)	<0.0001	2.18 (1.55-3.10)	<0.0001
Model 2	1.96 (1.38-2.82)	0.0002	2.28 (1.61-3.30)	<0.0001	2.31 (1.62-3.34)	<0.0001
Screen-detected diabetes (n=503)	120		122		116	
Model 1	0.78 (0.60-1.00)	0.05	0.81 (0.63-1.04)	0.1	0.76 (0.59-0.98)	0.04
Model 2	0.81 (0.63-1.05)	0.12	0.86 (0.66-1.11)	0.25	0.82 (0.63-1.07)	0.14
Total diabetes (n=856)	214		224		223	
Model 1	1.07 (0.86-1.32)	0.56	1.15 (0.94-1.42)	0.18	1.14 (0.92-1.40)	0.23
Model 2	1.12 (0.91-1.39)	0.29	1.25 (1.01-1.55)	0.04	1.22 (0.98-1.52)	0.07

Mode l: crude odds ratio; Model 2: adjusted for age, education/AHLE, residence and geographical area

Table S4. Sex-specific unadjusted and adjusted odds ratios (95% CIs) for self-reported diabetes according to education in the CHARLS population regardless of availability of blood sample with comparison to illiterate group (n=15181)

**Diabetes**	**Education (ref: illiterate)**							
	**Literate**	**p value**	**Elementary school**	**p value**	**Middle school**	**p value**	**High school and above**	**p value**
Men (7430)	1347		1953		1958		1221	
Self-reported diabetes (389)	70		103		84		101	
Model 1	1.63 (1.07-2.54)	0.03	1.65 (1.11-2.53)	0.02	1.33 (0.88-2.05)	0.18	2.68 (1.79-4.10)	<0.0001
Model 2	1.65 (1.07-2.59)	0.03	1.65 (1.09-2.55)	0.02	1.28 (0.83-2.03)	0.28	1.89 (1.21-3.03)	0.007
Women (7751)	1367		1325		1178		690	
Self-reported diabetes (490)	88		93		88		46	
Model 1	1.19 (0.91-1.54)	0.21	1.30 (1.00-1.68)	0.05	1.39 (1.06-1.81)	0.01	1.23 (0.87-1.71)	0.23
Model 2	1.20 (0.91-1.58)	0.19	1.30 (0.98-1.70)	0.07	1.21 (0.89-1.65)	0.22	0.81 (0.54-1.20)	0.30

Mode l: crude odds ratio; Model 2: adjusted for age, education/AHLE, residence and geographical area

Table S5. Sex-specific unadjusted and adjusted odds ratios (95% CIs) for self-reported diabetes according to annual household living expenditure in the CHARLS population regardless of availability of blood sample with comparison to the lowest quartile (n=15181)

Self-reported diabetes	Annual household living expenditure (ref: Q1)					
	Q2	p value	Q3	p value	Q4	p value
Men (7430)	1861		1886		1864	
Self-reported diabetes (389)	79		120		135	
Model 1	1.42 (1.00-2.03)	0.05	2.18 (1.58-3.04)	<0.0001	2.50 (1.83-3.48)	<0.0001
Model 2	1.42 (1.00-2.03)	0.05	2.08 (1.50-2.92)	<0.0001	2.29 (1.65-3.22)	<0.0001
Women (7751)	1967		1927		1912	
Self-reported diabetes (490)	122		147		152	
Model 1	1.80 (1.33-2.44)	0.0001	2.25 (1.68-3.03)	<0.0001	2.35 (1.76-3.16)	<0.0001
Model 2	1.90 (1.41-2.59)	<0.0001	2.38 (1.77-3.22)	<0.0001	2.47 (1.83-3.36)	<0.0001

Mode l: crude odds ratio; Model 2: adjusted for age, education/AHLE, residence and geographical area

Table S6. Characteristics of participants included and excluded in the primary analysis

Characteristics	Participants included (n=10100)	Participants excluded (n=7608)	P value
Age (years)			<0.0001
45-54	3416 (33.8)	3035 (40.0)	
55-64	3893 (38.5)	2553 (33.7)	
65-74	1968 (19.5)	1260 (16.6)	
≥75	823 (8.1)	734 (9.7)	
Mean (SD)	59.4 (9.6)	58.5 (10.9)	<0.0001
Sex			
Men	4791 (47.4)	3680 (48.5)	0.18
Self-reported diabetes			
Yes	605 (6.0)	388 (5.3)	0.06
Education			<0.0001
Illiterate	2914 (28.9)	1889 (25.0)	
Literate	1868 (18.5)	1272 (16.8)	
Elementary school	2223 (22.0)	1589 (21.0)	
Middle school	2034 (20.1)	1618 (21.4)	
High school and above	1061 (10.5)	1185 (15.7)	
AHLE			0.94
Q1	2479 (24.5)	1391 (24.8)	
Q2	2553 (25.3)	1395 (24.9)	
Q3	2528 (25.0)	1414 (25.2)	
Q4	2540 (25.1)	1400 (25.0)	
Residence			<0.0001
Rural	6513 (64.5)	4024 (52.9)	
Migrants	2008 (19.9)	1591 (20.9)	
Urban	1579 (15.6)	1990 (26.2)	
Geographical area			<0.0001
West	3453 (34.2)	2310 (30.4)	
Central and northeast	3766 (37.3)	2565 (33.7)	
East	2881 (28.5)	2730 (35.9)	

Values are N (%); missing data for participants excluded in the analysis were 27, 15, 299, 55, 2008, 3, and 3 for age, sex, self-reported diabetes, education, AHLE, residence, and geographical area, respectively.
